# Analysis of Colostrum and Udder Skin Swabs from a Dairy Goat Herd in Germany regarding the Occurrence of *Mycobacterium avium* Subsp. *paratuberculosis*

**DOI:** 10.3390/ani12141779

**Published:** 2022-07-11

**Authors:** Chris Pickrodt, Karsten Donat, Udo Moog, Heike Köhler

**Affiliations:** 1 Institute of Molecular Pathogenesis, Friedrich-Loeffler-Institut, Federal Research Institute for Animal Health, Naumburger Straße 96a, 07743 Jena, Germany; chris.pickrodt@fli.de; 2 Thuringian Animal Diseases Fund, Victor-Goerttler-Straße 4, 07745 Jena, Germany; kdonat@thtsk.de (K.D.); umoog@thtsk.de (U.M.); 3 Clinic for Obstretics, Gynaecology and Andrology for Large and Small Animal Science with Veterinary Ambulance, Justus Liebig University Giessen, Frankfurter Straße 106, 35392 Giessen, Germany

**Keywords:** *Mycobacterium avium* subspecies *paratuberculosis* (MAP), goats, colostrum, udder skin swab

## Abstract

**Simple Summary:**

The analysis of transmission routes for paratuberculosis as well as their prevention are critical for effective disease control. The aim of the present study was to assess the importance of colostrum and the udder skin as routes for transmission of *Mycobacterium avium* subspecies *paratuberculosis* (MAP) within a dairy goat herd. Swabs of the udder skin and colostrum samples were collected from goats of a paratuberculosis-infected herd during lambing season and analyzed for MAP DNA. Additionally, cultivation of the swabs was performed. MAP could not be detected in the colostrum. A low number of udder skin swabs was positive for MAP DNA but no bacteria could be cultured. Because the detection methods are imperfect, the occurrence of MAP in colostrum or on the udder skin can never be completely excluded. Therefore, present recommendations concerning colostrum and youngstock management should still be re-evaluated, but applied in goat herds within a paratuberculosis control program until the role of colostrum and udder skin for within-herd transmission of MAP is further investigated.

**Abstract:**

Oral intake of *Mycobacterium avium* subspecies *paratuberculosis* (MAP) in first days of life is considered to be the main route of infection for paratuberculosis. This can be related to a direct contact to contaminated feces or feeding of MAP containing colostrum. Colostrum is believed to become contaminated either by lactogenic shedding or introduction of MAP from environmental sources. In this pilot study, the presence of MAP in individual and bulk colostrum samples from a paratuberculosis-infected, vaccinated dairy goat herd in Germany and the effect of udder skin disinfection on the MAP load of colostrum were examined. In order to distinguish between lactogenic shedding and fecal contamination, 49 udder skin swabs were cultivated on solid medium whereas 29 swabs were additionally analyzed by qPCR. qPCR was applied on 110 individual colostrum samples collected from 55 goats, one before and one after disinfection with a mycobactericidal disinfectant, and 14 bulk colostrum samples. MAP DNA was detected in 10.3% (3/29) of the swab samples, but no viable MAP was cultivated from any sample. These results indicate a low-level MAP contamination of the udder skin and colostrum of milking goats suggesting a low risk of MAP transmission via these routes.

## 1. Introduction

*Mycobacterium avium* subspecies *paratuberculosis* (MAP) causes a chronic wasting disease in ruminants known as paratuberculosis or Johne’s disease. Symptoms such as emaciation despite normal feed intake, diarrhea and a decrease in milk yield are observed partly after years of incubation. During the subclinical stage of the disease, the pathogen is already shed into the environment, primarily with the feces. Starting intermittent shedding frequency as well as intensity increases with disease progression [1,2,3]. Studies also revealed an increasing likelihood of a lactogenic shedding of MAP in animals showing clinical symptoms [4].

Infection occurs at a very young age, usually in the first days of life. This period is particularly relevant for disease control because susceptibility for the pathogen decreases with increasing age [5]. As the pathogen is most commonly ingested orally, MAP contaminated colostrum may serve as a source for earliest infection [1]. MAP was first detected in milk samples from clinically symptomatic cows in 1929 [6]. Further studies proved the presence in milk from asymptomatic cows [7] as well as in colostrum [8].

In contrast to bovine milk, studies about the prevalence of MAP in goat milk are rare. The percentage of DNA-positive samples varied considerably among studies, ranging from 1.94% to 37.74%. The results depended on the country where the study was conducted, which is probably associated with the locally common husbandry conditions. A high prevalence was found in India, whereas it was low in Brazil, Norway and the Netherlands [9]. Currently, there is no comparable study for German goat herds.

The presence of MAP in milk can be attributed on the one hand to lactogenic shedding or, otherwise, may result from external contamination occurring during or after milking [5,8,10]. Inappropriate milking or storage of the milk can lead to contamination with MAP containing feces or an introduction of the pathogen out of the farm environment. MAP has been detected in bedding, water, and dust samples from cattle and sheep farms in several studies [11,12,13]. In particular, herds with a high prevalence of MAP-shedding animals are at risk of intensive indoor spread [14]. Since MAP shows a long survival time in the environment [15], airborne spread or a transmission from burdened bedding over the udder skin into the milk during the milking process is conceivable. Diarrhea as a clinical symptom associated with heavy shedding of the pathogen may increase the contamination of the bedding and thus the risk of a transmission of MAP to the udder skin of other herd members as well.

Whether MAP can be transferred via the udder skin into the milk is not yet known. In part, this is due to the fact that no validated methods to determine the presence of MAP on the udder skin and in goat colostrum or milk are available.

This study focused on two different objectives. First, the prevalence of MAP on the udder skin and in the colostrum of dairy goats of a herd in Germany should be determined. As a carry-over of MAP from the farm environment via the udder skin into the colostrum is a conceivable route for contamination, the effect of a disinfection of the udder skin on the occurrence of MAP in the colostrum should be evaluated as well.

Validated diagnostic tools for the detection of MAP on the udder skin as well as in goat colostrum do not exist. Therefore, new methods for DNA extraction had to be developed and evaluated with respect to their limit of detection in combination with established kits for the molecular detection of MAP.

## 2. Materials and Methods

### 2.1. Animal Welfare and Legislation

This study was carried out in strict accordance with European and national laws for the care and use of animals. It was approved by the Animal Health and Welfare Unit of the Thuringian State Office for Consumer Protection (file reference: 2684-04-04-BFI-20-103). During the entire study every effort was made to minimize suffering.

### 2.2. Study Herd

Samples were collected from a commercial organic dairy herd of approximately 350 lactating goats in Thuringia, Germany, in March and May 2020, as well as in February and March 2021. Fecal shedding of MAP was detected by culture in 92 out of 307 (30.0%) of the herds’ goats in 2018. Subsequently, all adult goats and the female youngstock had been vaccinated with an inactivated vaccine (Gudair, CZ Vaccines, O Porriño, Spain). Further herd examinations in 2020 and 2021 revealed an apparent within-herd prevalence of 7.8% and 3.1% MAP shedders, respectively. Fecal shedding of each goat was repeatedly determined by fecal culture following a standardized protocol [16]. Goats with at least one positive culture result for MAP were considered as infected.

Rotational grazing was performed during the grazing season between May and November when goats are only driven into the barn for milking twice a day. The deep litter was removed before the end of the grazing season and new bedding material was added every day as necessary ensuring clean coat and udder skin. Dried off goats were grouped in separated pens approximately six weeks before lambing. The rotary milking parlor was located in the building but separated from the animal area by walls. Milking was performed without a prior cleaning of the udder or of the milking equipment within one milking time. The first three milk yields after lambing were obtained with a separate portable milking machine, and the milked colostrum of all goats during one milking time was collected in a bucket and fed as bulk colostrum to the goat kids.

Kids were raised at the farm for replacement. They were separated directly after birth and fed manually within the first five hours of life. After two further feedings of bulk colostrum, feeding was changed to an ad libitum supply of commercial milk replacer. During five days each of the lambing seasons in 2020 and 2021, every goat lambing within that time was sampled for the study regardless of the individual paratuberculosis status, body condition, age and number of parturitions. Samples were collected during the first milking time in the rotary milking parlor. In total, 110 individual colostrum samples and 49 swab samples from the udder skin as well as 14 bulk colostrum samples were obtained. In addition, udder skin swabs were taken from every goat of which individual colostrum samples were collected. From six goats, only colostrum was obtained. Four goats were sampled twice (in 2020 and in 2021); therefore, 55 individual samples were received from 51 goats. Five additional goats, not sampled individually, contributed to the bulk colostrum samples.

### 2.3. Detection of MAP Contamination of Udder Skin

#### 2.3.1. Evaluation of Sampling Method for Swab Samples

##### Preparation of Bacteria

A dilution series was prepared consisting of a MAP-isolate (20MA0472) from a fecal sample of a goat from the study herd. To classify the MAP strain type, the PCR according to Collins et al. [17] was performed revealing the affiliation to the cattle-type (C-type).

The isolate was first sub-cultivated on Herrold’s Egg Yolk Agar with Mycobactin J and Amphotericin, Nalidixic acid and Vancomycin (HEYM, Becton Dickinson, Heidelberg, Germany) and later transferred to Middlebrook 7H9 broth (Becton Dickinson, Heidelberg, Germany) with glycerol (Carl Roth GmbH, Karlsruhe, Germany), Middlebrook OACD enrichment and Mycobactin J (Becton Dickinson, Heidelberg, Germany) for further propagation at 37 °C. The suspensions optical density (OD) was measured at 580 nm and adjusted to 0.35 OD_580_, followed by 10-fold serial dilutions (10^−1^ to 10^−9^) in distilled water. In total, 100 µL of each dilution was plated on Middlebrook agar 7H10 (Becton Dickinson, Heidelberg, Germany) with glycerol (Carl Roth GmbH, Karlsruhe, Germany), Middlebrook OACD enrichment, Mycobactin J (Becton Dickinson, Heidelberg, Germany) and amphotericin B (Sigma Aldrich, Taufkirchen, Germany) to determine the bacterial counts of the suspension. Colony counts were obtained after 5 weeks of incubation at 37 °C.

##### Sampling Using Dry and Moistened Cotton Swabs

The udder skins of three adult goats from a paratuberculosis non-suspect herd were obtained after regular slaughter at a slaughterhouse. They were used to determine and compare the detection limit for MAP after sampling with dry and moistened cotton swabs. Remains of the glandular tissue were removed from the skins and 24 pieces of 10 cm^2^ each were cut out and fastened on polystyrene pads.

A total of 1 mL of the 10^−2^, 10^−4^, 10^−6^, 10^−8^ and 10^−9^ dilution as well as a distilled water control was evenly applied to four skin pieces each. After overnight drying, two of the four prepared skin pieces were sampled with dry sterile cotton swabs (Heinz Herenz Medizinalbedarf GmbH, Hamburg, Germany) and two pieces were sampled with sterile cotton swabs moistened with distilled water. Two swabs were gently rubbed over each skin piece. Sticks were shortened and both swabs were transferred together into a sterile tube and stored at −20 °C.

A second trial using only dry cotton swabs to increase the number of comparable attempts was performed. The abdominal skin of three 3-month-old goat kids from the same farm as the adult goats which underwent necropsy for a different study was transferred from the dissection hall to the laboratory. Hair and subcutaneous fat were removed. Five pieces of 10 cm^2^ each were cut out per skin and fastened on polystyrene pads. A dilution series from 10^−1^ to 10^−9^ was prepared and the bacterial count was determined as described above. An amount of 1 mL of the dilution steps was applied to the skin parts and dried overnight. The skin pieces were sampled with dry sterile cotton swabs (Heinz Herenz Medizinalbedarf GmbH, Hamburg Germany) the same way as described above.

##### Real-Time PCR (qPCR)

Each sample was analyzed by qPCR and culture. Therefore, swabs were thawed within the tube, 5.5 mL distilled water was added and the tube was placed in a shaking incubator for 48 h at 37 °C and 50 rpm. After vortexing, 500 µL of the liquid was transferred to a 1.5 mL tube and DNA extraction was performed using the QIAamp DNA Mini Kit (Qiagen, Hilden, Germany). The DNA extracts were analyzed in duplicate with the ADIAVET PARATB REAL TIME PCR kit (Adiagene, Bio-X Diagnostics S.A., Rochefort, Belgium), a detection method based on the amplification of the insertion element IS900 of MAP, following instructions provided by the manufacturer. A reaction volume of 20 µL and 5 µL DNA template was used. A negative (nuclease-free water) and a positive control (provided within the kit) were included in each qPCR run. In total, 1 µL exogenous internal control of amplification (EPC-Amp) was added to each sample. qPCR was run on the QuantStudio 5 (Thermo Fisher Scientific, Langenselbold, Germany). The reaction conditions were 2 min at 45 °C, 10 min at 95 °C, followed by 45 cycles of 15 s at 95 °C and 30 s at 60 °C.

Data were analyzed using the QuantStudio Design & Analysis Software, v1.5.1 (Life Technologies, Carlsbad, CA, USA). Mean cycle threshold (C_t_) values of the duplicates were determined. Samples with C_t_ values ≤40.0 were considered positive. Values above the detection limit of 40.0 were considered negative and defined as 40.01 for analysis.

##### Bacterial Cultivation

A total of 5 mL of 1.5% Hexadecylpyridinium chloride monohydrate solution (HPC, Sigma Aldrich, Taufkirchen, Germany) was added to the remaining fluid and swabs in the tube. After shaking for 20 min at 200 rpm on a platform shaker, the tubes with the swabs were stored in the dark for 48 h at room temperature. Swabs were removed, and the supernatants were discarded to 5 mL and vortexed. Then, 250 µL was transferred on each of three slopes of HEYM and incubated at 37 °C for 16 weeks. Examination of bacterial growth was conducted every second week starting after 42 days.

#### 2.3.2. Udder Swab Samples Collected in the Study Herd

##### Animals

As described above, 49 milking goats were sampled directly after parturition during the first milking time on the collection days in the lambing periods in 2020 (number of samples (*n*) = 20) and 2021 (*n* = 29). The infection status of every participating goat was repeatedly determined before and during the study by fecal culture following a standardized protocol [16]. Goats with at least one positive culture result for MAP were considered as infected. This applied for five of the 49 samples (10.2%). Detailed information about the origin of the samples is provided in Table 1.

##### Sampling

Swab samples (*n* = 49) of the udder skin of the goats were taken with sterile cotton swabs (Heinz Herenz Medizinalbedarf GmbH, Hamburg, Germany) while standing in the rotary milking parlor. Two dry swabs were successively rubbed over both udder halves and the teats of the goat. After shortening the sticks, both swabs were stored in a sterile tube, frozen at −20 °C and transferred to the laboratory.

The samples from 2021 were analyzed by culture and qPCR the same way as described above. Samples from 2020 were analyzed by culture only. Deviating from the procedure applied in 2021, swabs were thawed within the tube and 5 mL of 0.75% HPC instead of 5 mL of 1.5% HPC were added to achieve a final concentration of 0.75% HPC in both years. The subsequent steps of bacterial cultivation remained unchanged.

### 2.4. Detection of MAP in Goat Colostrum

#### 2.4.1. Evaluation of Detection Method for MAP in Goat Colostrum

After adjusting the bacterial suspension to 0.35 OD_580_, 10-fold serial dilutions (10^−1^ to 10^−8^) of MAP isolate 20MA0472 were prepared in bulk goat colostrum from the studied herd as well as in distilled water which was analyzed for bacterial counting the same way as described above. Two times 10 mL of every dilution were processed directly after preparation using the ADIAPURE PARATB MILK kit (Adiagene, Bio-X Diagnostics S.A., Rochefort, Belgium) according to manufacturers’ instructions to extract MAP DNA based on immunomagnetic separation (IMS). Subsequently, the extracts were analyzed in duplicate with the ADIAVET PARATB REAL TIME PCR kit (Adiagene, Bio-X Diagnostics S.A., Rochefort, Belgium) under the same conditions described above and mean C_t_ values were determined. Samples with C_t_ values ≤40.0 were considered positive. Values above the detection limit of 40.0 were considered negative and defined as 40.01 for analysis.

The experiment was performed twice, each time with newly prepared spiked colostrum.

#### 2.4.2. Colostrum Samples Collected in the Study Herd

##### Animals

**Figure 1:**
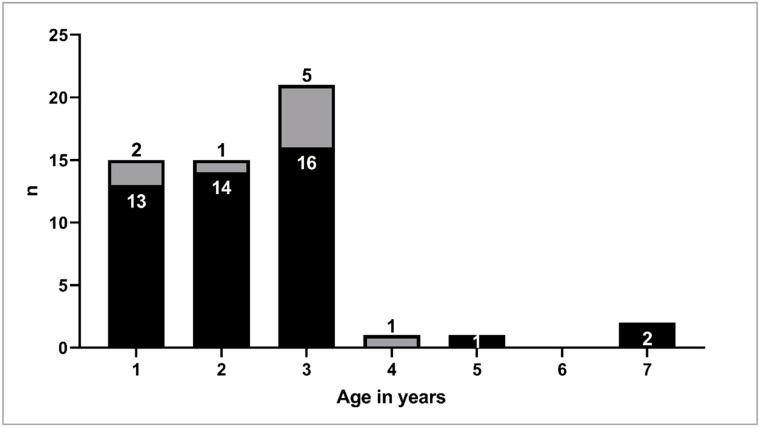
Age distribution and *Mycobacterium avium* subspecies *paratuberculosis* (MAP) status of the sampled goats within the study (*n* = 55). MAP-negative (black, *n* = 46), MAP-positive (gray, *n* = 9).

As described above, 55 milking goats were sampled directly after parturition during the first milking time. Bulk colostrum consisted of the milk from two to twelve goats. The age of the individually sampled goats varied between one and seven years. None of the goats showed clinical signs of paratuberculosis. Nine of the 55 individual colostrum sample sets (16.4%) originated from goats with at least one positive fecal culture result during regular herd examinations since 2018. A bulk colostrum sample was defined as originating from MAP-positive goats if at least one of the contributing goats was classified as MAP-positive. Seven of 14 collected bulks (50.0%) contained the milk of one (3/7; 42.9%) or two (4/7; 57.1%) infected goats. Detailed information about the origin of the samples is provided in Table 1 and Figure 1.

##### Sampling

Two colostrum samples (*n* = 110) of approximately 30 mL each were obtained per animal on the day of lambing from both udder halves while standing in the rotary milking parlor. The first one was taken from the non-disinfected udder followed by a second sample after cleaning with a mycobactericidal disinfectant (Sterilium Tissue, BODE CHEMIE GmbH, Hamburg, Germany) and discard of the first milk. Furthermore, bulk colostrum samples (*n* = 14) were collected for analysis. A total of 50 mL of the separate bulks were bottled out of the collection bucket into a vial after the end of the milking time. All samples were frozen at −20 °C directly after collection, transferred to the laboratory and kept at this temperature until further processing.

Samples were thawed at room temperature. In total, 10 mL of each colostrum sample was processed using the ADIAPURE PARATB MILK kit (Adiagene, Bio-X Diagnostics S.A., Rochefort, Belgium). DNA extracts were examined in duplicate by the ADIAVET PARATB REAL TIME PCR kit (Adiagene, Bio-X Diagnostics S.A., Rochefort, Belgium) following instructions provided by the manufacturer. Mean C_t_ values of the duplicates were determined. Samples with C_t_ values ≤40.0 were considered positive. Values above the detection limit of 40.0 were considered negative and defined as 40.01 for analysis.

### 2.5. Data Analysis

Data analysis was performed using Microsoft Office Excel version 2019 (Microsoft Corporation, Redmond, WA, USA). Figures were created using GraphPad Prism software version 9.1.3 (GraphPad Software, San Diego, CA, USA).

## 3. Results

### 3.1. MAP Contamination of Udder Skin

#### 3.1.1. Detection Limit of MAP in Swab Samples

The colony counts revealed total MAP numbers in the OD-adjusted bacterial suspensions of 1.6 × 10^7^ cfu/mL for the comparison of dry and moistened swabs (trial 1) and 2.1 × 10^7^ cfu/mL for the following experiment with dry swabs only (trial 2).

**Figure 2:**
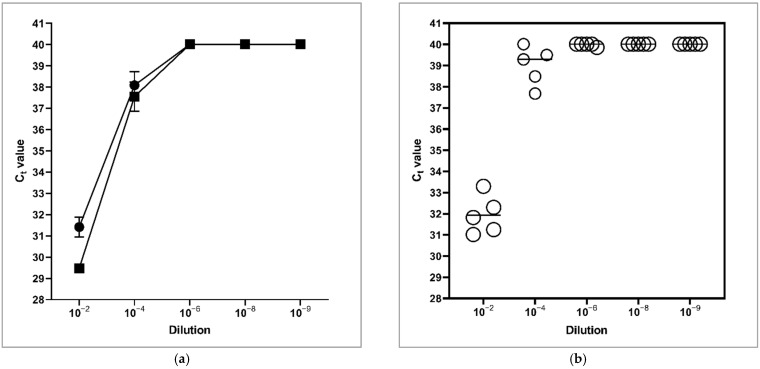
C_t_ values obtained for serial dilutions of *Mycobacterium avium* subspecies *paratuberculosis* on udder skin by sampling with cotton swabs after DNA extraction using the QIAamp DNA Mini Kit (Qiagen, Hilden, Germany) and the ADIAVET PARATB REAL TIME PCR kit (Adiagene, Bio-X Diagnostics S.A., Rochefort, Belgium). (**a**) Comparison of dry (⬤) and moistened swabs (◼) expressed as means ± SD; (**b**) Comparison of dry swabs from trial 1 and 2. Each dot represents an individual sample. Mean C_t_ values of the dilutions are denoted by horizontal lines.

C_t_ values obtained from dry swabs were higher than from moistened swabs in the 10^2^ dilution (31.4 vs. 29.8). This difference disappeared in the 10^−4^ dilution (38.1 vs. 37.6) (Figure 2a). Apart from one detection in the 10^−6^ dilution of the MAP-suspension, C_t_ values were regularly measured up to the 10^−4^ dilution corresponding to a MAP load of 1.6–2.1 × 10^2^ cfu/cm^2^ of skin when dry cotton swabs were used (Figure 2b).

MAP was cultivated out of all dry (5/5, 100%) and all moistened (2/2, 100%) swab samples of the 10^−2^ dilution within six weeks after inoculation and out of 3/5 (60%) of the dry swab samples of the 10^−4^ dilution within six to eight weeks after inoculation. MAP was not isolated from all other dry and moistened swabs of the dilutions 10^−4^ to 10^−9^. Hence, the detection limit by culture for viable MAP on the udder skin of goats using dry swabs is between 10^2^ and 10^4^ cfu/cm^2^.

#### 3.1.2. Detection of MAP on the Udder Skin of Goats from a MAP-Positive Dairy Herd

MAP could not be cultivated from any of the processed swab samples (*n* = 49). C_t_ values of 35.8, 38.7 and 39.5, respectively, were detected in three of the 29 swab samples (10.3%), which were analyzed by qPCR. All positive samples originated from MAP-negative goats.

### 3.2. MAP Contamination of Colostrum

#### 3.2.1. Detection Limit of MAP in Spiked Colostrum Samples

**Figure 3:**
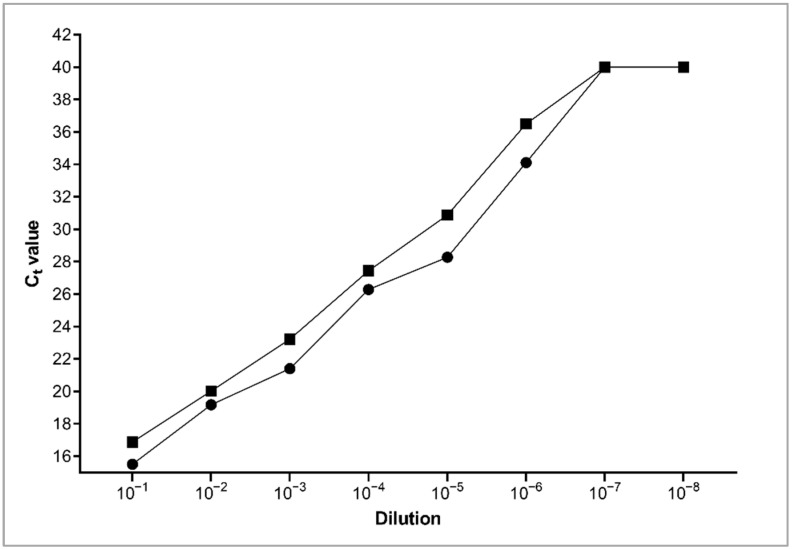
C_t_ values obtained for serial dilutions of *Mycobacterium avium* subspecies *paratuberculosis* in caprine colostrum after DNA extraction using the ADIAPURE PARATB MILK kit and the ADIAVET PARATB REAL TIME PCR kit (Adiagene, Bio-X Diagnostics S.A., Rochefort, Belgium). Trial 1 (◼) and trial 2 (⬤).

The colony counts revealed total MAP numbers in the OD-adjusted bacterial suspensions of 7.9 × 10^7^ cfu/mL for the first trial and 3.3 × 10^7^ cfu/mL for the second trial. The lowest dilution in spiked colostrum giving a detectable qPCR signal was about 10^−6^ (Figure 3), corresponding to a MAP load of 33–79 cfu/mL. Lower bacterial counts were not detectable. Hence, the detection limit for MAP DNA in goat colostrum is between 33 and 79 cfu/mL.

#### 3.2.2. Detection of MAP in Colostrum of Goats from a MAP-Positive Dairy Herd

MAP could be detected neither in one of the 110 colostrum samples from 55 different goats nor in the 14 bulk colostrum samples by qPCR. All individual colostrum samples obtained before and after a cleaning of the udder with a mycobactericidal disinfectant were negative.

## 4. Discussion

This study analyzed colostrum samples and swabs of the udder skin from milking goats of a paratuberculosis-positive herd to determine the prevalence of MAP on the udder skin and in the colostrum. Furthermore, the effect of a disinfection of the udder on the occurrence of MAP in the colostrum was evaluated to ascertain if this measure can prevent a possible carry over of MAP out of the farm environment via the udder skin into the colostrum.

Assessment of MAP prevalence on the udder skin as a source of contamination of colostrum or milk was the first part of the study. For the sampling, a new protocol based on wiping sterile cotton swabs over the udder skin was developed and tested for its sensitivity concerning the detection of MAP DNA by qPCR and viable MAP by culture. Furthermore, we examined if dry or moistened swabs achieved better results. MAP DNA could be detected for amounts of 1.6 to 2.1 × 10^2^ cfu/cm^2^ and cultivation was successful down to bacterial loads between 10^2^ and 10^4^ cfu/cm^2^. The direct comparison between dry and moistened cotton swabs revealed similar C_t_ values for DNA detection. Because of the comparable analytic sensitivity of both variants, it was decided to use dry swabs for the sampling of the goats in this study. This would also prevent a potential cross contamination using the same fluid to moisten different swabs during sampling at the farm.

In contrast to Pithua et al. [10], who detected MAP DNA in 60% of teat swabs of milking cows from a herd with paratuberculosis, in the present study, MAP could only be detected in 10.3% (3/29) of the samples. The high C_t_ values in combination with the completely negative culture results indicate a low contamination of the udder skin of dairy goats from the study herd with MAP. All positive samples originated from MAP-negative goats. This fact and the negative results of five MAP-positive animals lead to the assumption that the occurrence of MAP on the udder skin is independent from the fecal MAP status of the individual goat. However, the small number of samples allows no conclusion about the significance of this finding.

MAP DNA was not detected in the colostrum samples of the goats before and after disinfection of the udder skin. These results are in contrast to the common assumption that MAP can be shed into colostrum. For dairy cows, evidence for that transmission route is provided by some studies [4,8]. External contamination of colostrum by feces is discussed as an alternative source for MAP introduction [5,8,10]. Because of the limited number of studies concerning MAP detection in colostrum in small ruminants [9,18] it is difficult to rate our results in the context of the current knowledge concerning this field of research.

The fact that MAP DNA was not found in the 55 individual colostrum samples obtained aseptically after disinfection of the udder provided evidence that lactogenic shedding did not occur in the goats of our study. However, it cannot be ruled out that the MAP load of the colostrum, if any, was below the detection limit of our method, which amounted to 33–79 cfu/mL. As the commercial test kit for the detection of MAP used in this study was only validated for bovine milk, the detection limit for goat colostrum had to be determined in advance. Analysis of milk samples is hampered by the high content of protein and fat contributing to a low sensitivity of direct PCR without any further preparation of the sample [19]. This situation worsens when colostrum has to be examined due to its characteristic composition. Colostrum contains a higher proportion of fat, protein and somatic cells than milk [4,20]. Sample preparation by IMS has been proven to be an effective way to lower the detection limit of MAP in milk samples [19]. The specific method of IMS enables the separation of the desired bacteria from other PCR-inhibiting contents and their concentration. Using colostrum from the study herd, a limit of detection between approximately 33 and 79 cfu/mL was revealed. This is comparable to the limits of an IMS protocol developed by Grant [21] for the analysis of bovine milk samples with 20 cfu/mL. Therefore, a serious failure of the detection method is unlikely.

As shown by another study, an interference between the antibodies used for IMS and host antibodies induced by vaccination is unlikely. Djønne et al. [22] analyzed milk samples from goats at different points of lactation with a self-designed IMS method. There was no difference between vaccinated and unvaccinated animals concerning the percentage of positive samples.

Currently there is no certain knowledge about the amount of MAP shed into milk or the colostrum [23]. For infected but asymptomatic cows, a number of 2–8 cfu per 50 mL milk was suggested [7]. Stabel et al. [4] showed a relation between clinical disease stage and probability of lactogenic shedding of MAP. In total, 12.6% of analyzed milk samples from cows with subclinical and 49.2% from cows with clinical paratuberculosis were positive using direct PCR. Furthermore, the amount of MAP was higher in samples from clinically affected cows. An association between the occurrence of lactogenic and the intensity of fecal shedding in cows was also observed [8]. In that study, MAP was more often detected in the colostrum of high fecal shedders than of light shedders, but colostrum from two non-shedding cows was also positive for MAP. As there was not one goat in the clinical stage of paratuberculosis and just 16.4% (9/55) were diagnosed as infected with MAP in our study, this may have influenced the allover negative results. Furthermore, the whole study herd was vaccinated against paratuberculosis. The goal of this measure was to reduce MAP-shedding and to protract the clinical stage of the disease [2]. The probability to detect MAP in colostrum samples resulting from lactogenic shedding of the pathogen was therefore lower as if the study would have been carried out in a herd with a higher disease prevalence or if just infected goats or animals showing clinical symptoms of paratuberculosis would have been sampled. Additionally, 30 out of the 55 examined goats (54.5%) were two years or younger (Figure 1). As the severity of the disease increases with age [1], as a consequence, the likelihood of lactogenic shedding is low, as younger goats are more likely not to shed MAP into the colostrum. This assumption is supported by findings of Djønne et al. [22], who analyzed milk samples from goats of different ages and revealed that older goats showed more often positive results than younger goats. Nevertheless, the goats selected for this investigation represent the age profile of the study herd which is typical for a commercial dairy herd. In consideration of the limitations resulting from the age as well as the corresponding stage of disease and amount of shedding, we decided not to apply any selection criteria to the sampled goats to avoid a selection bias.

Lactogenic shedding of MAP is assumed to be discontinuous as has been shown for fecal shedding [24,25]. During the dry period, MAP is possibly transferred within macrophages from the intestine over the lymphatic system to the udder. This can lead to an accumulation in the colostrum because macrophages are the most common mononuclear cells in the first milk [26,27]. This assumption was strengthened by studies where cows were sampled several times during lactation and more colostrum than milk samples were positive for MAP [4,8]. Similar results were observed for the lactogenic shedding in asymptomatic sheep. However, whereas six out of 20 examined sheep showed lactogenic shedding approximately ten days after parturition, no positive milk sample was obtained from goats (0/9) at this point [24]. This matches the negative results of the analyzed goat colostrum samples in our study. MAP-infected goats in the subclinical stage of disease appear to have a lower likelihood for MAP-positive colostrum than other ruminant species. In a study from The Netherlands, MAP could not be detected in any colostrum sample from animals in the subclinical stage of disease, whereas it was detected in four percent of the milk samples obtained later during lactation [18]. MAP was also detected in milk samples from dairy goats during different timepoints of lactation in a study from India [25]. Although the number of relevant studies is very small, there seems to be a genus difference which cannot be explained at the moment. Further studies are necessary to prove this difference and to elucidate the pathogenetic mechanisms behind it.

In addition to the individual colostrum samples, the analyzed bulk colostrum had also tested negative for the occurrence of MAP. These results were not surprising as the bulk colostrum was composed of the colostrum of two to twelve goats, where MAP was not detected in the individual samples. Even if MAP was detected in a small number of individual samples, the dilution effect resulting from pooling of different colostrum would have led to a further reduction of MAP per mL, possibly below the detection limit. Bulk colostrum in the study herd is prepared by milking individual goats into a container using a separate milking machine. This practice does not seem to be a source of contamination of the colostrum with MAP from the environment.

Due to the detection limit of 33–79 cfu/mL, the negative bulk colostrum samples can contain a small number of MAP, as already discussed. If this amount per mL is summed up to the recommended amount of colostrum of about 500 mL that the kid should receive within the first day of life, this can cause a total MAP intake of up to 10^4^ cfu. Compared to an assumed infectious dose of 50 to 1000 cfu [26], an infection remains possible. This underlines the necessity of good colostrum management in the herd.

Despite the missing detection of MAP in the analyzed colostrum samples, general recommendations concerning youngstock rearing in paratuberculosis-infected herds should be applied. This includes the feeding of colostrum only from MAP-negative goats. Furthermore, the feeding of bulk colostrum is critical and should be avoided wherever possible. This may reduce the potential spread of even low numbers of MAP to several kids in herds with a high prevalence of MAP-shedding goats. Moreover, hygienic collection, storage and feeding is the basis for a good kid health in general. This can also include a preventive disinfection or cleaning of the udder before milking colostrum to minimize contamination with MAP as well as other bacteria.

In the present study, the assumed transfer of MAP over the udder skin into the colostrum could not be proven because most of the udder skin swabs were negative and MAP could not be detected in the colostrum samples before disinfection. Nevertheless, this finding remains a hypothesis and should be further examined. The presumably higher prevalence of MAP on the udder skin of dairy cows in comparison to goats suggests performing such a study in cattle as well.

## 5. Conclusions

MAP DNA could not be detected in colostrum samples from milking goats of a paratuberculosis-positive vaccinated herd. Despite contamination of the farm environment due to fecal shedders, the transmission of MAP onto the udder skin is low. The results suggest a minor risk of MAP transmission into the colostrum from the udder skin during the milking process. These findings stand in contrast to results from dairy cows where MAP detection in both sample types has been reported. As this pilot study only analyzed a small number of samples from one farm, further studies in dairy goat herds have to be conducted to confirm reproducibility of the presented results, preferably using the methods evaluated for this study to ensure comparability.

Common recommendations concerning colostrum and youngstock management for herds of small ruminants are often derived from cattle. With respect to the assumed differences between these genera concerning the presence of MAP in colostrum, recommendations regarding hygiene improvement for the control of paratuberculosis in small ruminants should be re-evaluated.

## Figures and Tables

**Table 1:** Type and number of samples from *Mycobacterium avium* subspecies *paratuberculosis* (MAP)-positive and negative goats within the study. A bulk colostrum sample was defined as originating from MAP-positive goats if at least one of the contributing goats was classified as MAP-positive.

Sample Classification	Number of MAP-Positive Goats	Number of MAP-Negative Goats	Total
udder swab	5	44	49
individual colostrum from non-disinfected udder	9	46	55
individual colostrum from disinfected udder	9	46	55
bulk colostrum	7	7	14

## Data Availability

The datasets analyzed during the current study are available from the corresponding author on reasonable request.

## References

[1] Windsor P.A. (2015). Paratuberculosis in sheep and goats. Vet. Microbiol..

[2] Whittington R., Donat K., Weber M.F., Kelton D., Nielsen S.S., Eisenberg S., Arrigoni N., Juste R., Sáez J.L., Dhand N. (2019). Control of paratuberculosis: Who, why and how. A review of 48 countries. BMC Vet. Res..

[3] Collins P., Davies D., Matthews P. (1984). Mycobacterial infection in goats: Diagnosis and pathogenicity of the organism. Br. Veter. J..

[4] Stabel J., Bradner L., Robbe-Austerman S., Beitz D. (2014). Clinical disease and stage of lactation influence shedding of *Mycobacterium avium* subspecies *paratuberculosis* into milk and colostrum of naturally infected dairy cows. J. Dairy Sci..

[5] Windsor P.A., Whittington R.J. (2010). Evidence for age susceptibility of cattle to Johne’s disease. Veter J..

[6] Alexejeff-Goloff N.A. (1929). Zur Frage der Pathogenese und Bazillenausscheidung bei Rinderparatuberkulose. Zeits. Infekt. Krank. Haus..

[7] Sweeney R.W., Whitlock R.H., Rosenberger A.E. (1992). Mycobacterium paratuberculosis cultured from milk and supramammary lymph nodes of infected asymptomatic cows. J. Clin. Microbiol..

[8] Streeter R.N., Hoffsis G.F., Bech-Nielsen S., Shulaw W.P., Rings D.M. (1995). Isolation of Mycobacterium paratuberculosis from colostrum and milk of subclinically infected cows. Am. J. Vet. Res..

[9] Roberto J.P.d.L., Limeira C.H., Barnabé N.N.D.C., Soares R.R., Silva M.L.C.R., Gomes A.A.D.B., Higino S.S.D.S., de Azevedo S.S., Alves C.J. (2021). Antibody detection and molecular analysis for Mycobacterium avium subspecies paratuberculosis (MAP) in goat milk: Systematic review and meta-analysis. Res. Vet. Sci..

[10] Pithua P., Wells S.J., Godden S.M., Stabel J.R. (2011). Evaluation of the association between fecal excretion of *Mycobacterium avium* subsp *paratuberculosisand* detection in colostrum and on teat skin surfaces of dairy cows. J. Am. Vet. Med. Assoc..

[11] Lombard J., Wagner B., Smith R., McCluskey B., Harris B., Payeur J., Garry F., Salman M. (2006). Evaluation of Environmental Sampling and Culture to Determine *Mycobacterium avium* subspecies *paratuberculosis* Distribution and Herd Infection Status on US Dairy Operations. J. Dairy Sci..

[12] Whittington R.J., Marsh I.B., Taylor P.J., Marshall D.J., Taragel C., Reddacliff L.A. (2003). Isolation of *Mycobacterium avium* subsp *paratuberculosis* from environmental samples collected from farms before and after destocking sheep with paratuberculosis. Aust. Vet. J..

[13] Eisenberg S., Nielen M., Santema W., Houwers D., Heederik D., Koets A. (2010). Detection of spatial and temporal spread of *Mycobacterium avium* subsp. *paratuberculosis* in the environment of a cattle farm through bio-aerosols. Vet. Microbiol..

[14] Donat K., Schau U., Soschinka A. (2011). Identifizierung von mit Mycobacterium avium ssp. paratuberculosis (MAP) infizierten Milchviehbeständen mithilfe von Umgebungskotproben. Berl. Münch. Tierärztl. Wochenschr..

[15] Whittington R.J., Marshall D.J., Nicholls P.J., Marsh I.B., Reddacliff L.A. (2004). Survival and Dormancy of *Mycobacterium avium* subsp. *paratuberculosis* in the Environment. Appl. Environ. Microbiol..

[16] Friedrich-Loeffler-Institut Paratuberkulose: Amtliche Methode und Falldefinition. https://www.openagrar.de/receive/openagrar_mods_00058039.

[17] Collins D.M., De Zoete M., Cavaignac S.M. (2002). *Mycobacterium avium* subsp. *paratuberculosis* Strains from Cattle and Sheep Can Be Distinguished by a PCR Test Based on a Novel DNA Sequence Difference. J. Clin. Microbiol..

[18] Lievaart-Peterson K., Luttikholt S., Gonggrijp M., Ruuls R., Ravesloot L., Koets A.P. (2019). *Mycobacterium avium* Subspecies *paratuberculosis* DNA and Antibodies in Dairy Goat Colostrum and Milk. Vet. Sci..

[19] Husakova M., Dziedzinska R., Slana I. (2017). Magnetic Separation Methods for the Detection of *Mycobacterium avium* subsp. *paratuberculosis* in Various Types of Matrices: A Review. BioMed Res. Int..

[20] Sánchez-Macías D., Moreno-Indias I., Castro N., Morales-delaNuez A., Argüello A. (2014). From goat colostrum to milk: Physical, chemical, and immune evolution from partum to 90 days postpartum. J. Dairy Sci..

[21] Grant I. (2000). Improved detection of *Mycobacterium avium* subsp. *paratuberculosis* in milk by immunomagnetic PCR. Vet. Microbiol..

[22] Djønne B., Jensen M., Grant I., Holstad G. (2003). Detection by immunomagnetic PCR of *Mycobacterium avium* subsp. *paratuberculosis* in milk from dairy goats in Norway. Vet. Microbiol..

[23] Barkema H.W., Orsel K., Nielsen S.S., Koets A.P., Rutten V.P.M.G., Bannantine J.P., Keefe G.P., Kelton D.F., Wells S.J., Whittington R.J. (2018). Knowledge gaps that hamper prevention and control of *Mycobacterium avium* subspecies *paratuberculosis* infection. Transbound. Emerg. Dis..

[24] Nebbia P., Robino P., Zoppi S., De Meneghi D. (2006). Detection and excretion pattern of *Mycobacterium avium* subspecies *paratuberculosis* in milk of asymptomatic sheep and goats by Nested-PCR. Small Rumin. Res..

[25] Singh M., Gupta S., Chaubey K.K., Singh S.V., Sohal J.S. (2019). Profiling of *Mycobacterium avium* subspecies *paratuberculosis* in the milk of lactating goats using antigen-antibody based assays. Comp. Immunol. Microbiol. Infect. Dis..

[26] Chiodini R.J. (1996). Immunology: Resistance to Paratuberculosis. Vet. Clin. North Am. Food Anim. Pract..

[27] Hurley D.J., Kensinger M.H., Mastro A.M., Wilson R.A. (1990). An evaluation of the mononuclear cells eerived from bovine mammary gland dry secretions using leukocyte antigen specific monoclonal antibodies, light scattering properties and non-specific esterase staining. Vet. Immunol. Immunopathol..

